# Surgical Treatment Following Stent Angioplasty for High-Risk Neonates With Critical Coarctation of the Aorta

**DOI:** 10.1177/21501351221099933

**Published:** 2022-06-25

**Authors:** Philippe Grieshaber, Moritz Merbecks, Christoph Jaschinski, Elizabeth Fonseca, Raoul Arnold, Matthias Karck, Matthias Gorenflo, Tsvetomir Loukanov

**Affiliations:** 1 Department of Cardiac Surgery, 27178 University Hospital Heidelberg, Heidelberg, Germany; 2 Department of Pediatric Cardiology, 27178 University Hospital Heidelberg, Heidelberg, Germany

**Keywords:** aortic coarctation, stents, cardiac catheterization, thoracic surgery, pediatric emergency medicine

## Abstract

**Background:**

Neonatal coarctation of the aorta (CoA) is primarily treated by surgical repair. However, under certain high-risk constellations, initial stent angioplasty may be considered followed by surgical repair. We report our experience with this staged approach. **Methods:** All patients undergoing surgical CoA repair following prior stenting at our institution between January 2011 and December 2019 were included in this retrospective analysis. The patients were classified to be at high risk because of cardiogenic shock, associated complex cardiac malformations, neonatal infection, necrotizing enterocolitis, and extracardiac conditions, respectively. Outcomes were analyzed and compared with neonates who underwent surgical CoA repair without prior stenting in the same observation period.

**Results:**

Twenty-six neonates received stent implantation at a median age of 20 days (IQR 9-33 days). Subsequent surgical repair was conducted at an age of 4.2 months (IQR 3.2-6.1 months) with a median body weight of 5.6 kg (IQR 4.5-6.5 kg). Cardiopulmonary bypass was applied in 96% of cases. Extended end-to-end anastomosis was possible in 11 patients. Extended reconstruction with patch material was necessary in the remaining patients. One fatality (3.8%) occurred 33 days postoperatively. At a median follow-up of 5.2 years after initial stenting, all remaining patients were alive; 15/25 patients (60%) were free from re-intervention. Of note, re-intervention rates were comparable in neonates (n = 76) who were operated on with native CoA (28/74 patients; 38%; *P* = .67).

**Conclusions:**

Neonatal stent angioplasty for CoA results in increased complexity of the subsequent surgical repair. Nevertheless, this staged approach allows to bridge high-risk neonates to later surgical repair with reduced perioperative risk and acceptable midterm outcomes.

## Introduction

The primary therapeutic option for neonatal coarctation of the aorta (CoA) is surgical repair.^1^ Surgical repair comprises excision of the stenotic segment and either direct anastomosis of the aortic stumps or reconstruction of the aorta using patch material. Depending on the anatomical extent of CoA, the age of the patient and the presence of associated malformations (eg, hypoplastic aortic arch, septal defects, obstructions of the left ventricular outflow tract), the most frequent approaches for the surgical therapy are a left lateral thoracotomy and a median sternotomy with or without the use of cardiopulmonary bypass (CPB).^2^ Advances in interventional cardiology enabled interventional therapy of CoA using balloon angioplasty with or without stent implantation to be an additional therapeutic option.^3,4^ However, there are still limitations of interventional therapy (eg, high rates of re-CoA, re-interventions, lack of growth potential, incorporation of stent material) rendering it rather a palliative than definitive approach.^5‐7^ There are several clinical situations in which the risk of surgical CoA repair is increased (eg, cardiogenic shock, neonatal infections, necrotizing enterocolitis or other types of ischemic organ dysfunction, concomitant complex cardiac malformations).^8‐10^ In those patients, a staged therapeutic approach including initial palliative stenting of the CoA allowing the patients to stabilize and grow followed by surgical repair might be considered.^11,12^ However, a potentially relevant drawback of this strategy is increased complexity of the surgical repair, which has to include removal of the stent material and the adjacent aortic tissue. We report the results of this staged approach in a single center over a nine-year period.

## Patients and Methods

### Study Design

The present study was a retrospective single-center study.

### Ethics

The trial was designed and conducted in accordance with the Declaration of Helsinki. The patients’ parents gave consent to collection and analysis of their data for scientific purposes prior to discharge from the hospital. The local ethical committee approved the study.

### Study Population

All patients who underwent surgical repair for CoA following prior stenting in the neonatal period at our institution between January 2011 and December 2019 were identified from institutional patient records. Additionally, the surgical characteristics and postoperative outcomes of patients who underwent neonatal surgical CoA repair without prior stenting during the same time interval were analyzed. Long-term follow-up data were either extracted from institutional records or obtained via questionnaires sent to the patients’ parents or their family physicians.

### Decision Algorithm and Pre-interventional Management

All patients referred to our center were discussed immediately by an interdisciplinary heart team consisting of pediatric cardiologists and pediatric cardiac surgeons. If a patient was deemed to be at too high risk for surgical CoA repair, an emergent interventional CoA stent angioplasty was performed as soon as possible. Cardiogenic shock was assumed in patients with severely reduced left ventricular function (as assessed by echocardiography) and at least one clinical sign (cold, clammy, pale skin, tachypnea, tachycardia, oliguria). Pre-interventionally, all patients were stabilized with prostaglandin E1 infusion. Mechanical ventilation and inotropic support were added as needed. For patients who underwent stent angioplasty in external centers, the decision algorithm is reported to be identical except for the lack of a cardiac surgeon in the initial decision process.

### Stent Angioplasty

Stent angioplasty procedures were carried out under standardized conditions, as previously described.^12,13^ Briefly, a 4F sheeth was placed in the preferably right common femoral artery. A guidewire was advanced into the descending aorta and through the CoA into the ascending aorta. After introducing a pigtail catheter, pressures proximal and distal to the CoA were recorded. The position and length of the stenotic segment were determined and an appropriate stent chosen. The stent was then advanced into its target region using a balloon catheter and expanded using a manual pressure-controlled syringe. After angiographic control, additional dilatations were added as required. The pressure gradient at the end of the procedure was recorded again. After removal of the sheath, hemostasis was achieved using compression.

### Surgical Management

The surgical correction of CoA and stent extraction ± additional procedures were scheduled after the patients fully recovered from their preexisting critical situation that led to stent angioplasty of the CoA, as determined by an interdisciplinary heart team. The surgical access was either via a median sternotomy or a left lateral thoracotomy. Generally, the median sternotomy approach offering more options for arch reconstruction and correction of additional defects was favored, also with regard to the implanted stent that had to be extracted. The necessity of arch reconstruction was determined individually, depending on arch morphology and diameters. As a rule of thumb, the aortic arch was deemed to be hypoplastic, and arch reconstruction was considered to be necessary when the arch diameters deviated more than −2.5 SD (Z-score −2.5) from the expected value.^14,15^ The goals of surgical therapy were creation of an nonobstructive aortic arch and isthmus and complete removal of the stent material. If a median sternotomy approach was used, cardiopulmonary bypass was applied. The CoA repair ± arch reconstruction was conducted under selective cerebral perfusion. Deep hypothermic circulatory arrest was applied if distal aortic clamping was limited due to a far distal position of the stent. The stent material was completely removed, if possible. Depending on the anatomic situation, a direct anastomosis of the aorta was fashioned using polydioxanone (PDS) sutures. If the distance or arch anatomy were not suitable for direct anastomosis, the back wall of the aorta was anastomosed directly using an interdigitating technique and the anterior wall was augmented using patch material (autologous pericardium, xenopericardium, or pulmonary homograft). After completion of the reconstruction, the pressure gradients between the ascending aorta (or right radial artery) and femoral artery were directly measured (Supplemental Video 1).

### Endpoints

The primary endpoint of this analysis was in-hospital mortality. Secondary endpoints included the reduction of the pressure gradients, surgical complications (recurrent nerve paralysis, phrenic nerve paralysis, bleeding), long-term survival, and rate of re-interventions (surgical or interventional re-interventions), respectively. The staged strategy was assessed as a whole, that is, the stenting was considered to be the index procedure.

### Statistics

In this retrospective study, an inferential statistical analysis was performed using SPSS Version 26 (IBM) and GraphPad Prism version 6 software (GraphPad Software, Inc.) Patient characteristics: Data are shown as mean ± standard deviation (SD) unless stated otherwise.

Kaplan-Meier estimation was used for calculating long-term functions of survival and freedom from re-interventions for the staged approach and surgical repair of native CoA, respectively. The functions were compared between patients receiving different modes of therapy using the log-rank test. Group comparisons between patients with stented CoA and patients with native CoA were conducted using Fisher exact test, Student *t* test, or nonparametric tests, as appropriate. Statistical significance was assumed at a level of *P* < .05.

## Results

Of a total of 199 consecutive patients who underwent CoA repair with or without concomitant procedures between January 2011 and December 2019, 102 patients needed treatment in the neonatal period. Thereof, 26 patients underwent CoA stenting prior to surgery. Seventy-six patients underwent primary neonatal surgical repair. There was no patient during the observation period deemed to be at too high risk for any intervention. Thus, all CoA patients were offered treatment. The indications for the initial stenting were cardiogenic shock/severely impaired left ventricular function in 13 patients (50%), the presence of associated complex cardiac malformations in four patients (15%), neonatal sepsis in three patients (12%), necrotizing enterocolitis (stage IIB^16^) in patient (4%), and extracardiac conditions (asphyxia, intracerebral hemorrhage, pulmonary infection, diaphragmatic hernia) in four patients (15%), respectively. Of note, 4/26 patients were born and underwent stenting in a referring center without congenital cardiac surgical infrastructure. For one of these patients, the leading indication for stent placement was not revealable. Seven patients (27%) were endotracheally intubated and one patient (3.8%) was supported with venovenous extracorporeal membrane oxygenation prior to stenting (Table 1). There was no temporal trend in the frequency of stenting during the observation period.

**Table 1.** Baseline Characteristics of the 26 Patients Who Underwent Neonatal Stenting.

No.	Gender, age, weight	Diagnoses	Mechanical ventilaton pre-stent	Indication for coarctation stent	Diameter distal aortic arch (mm)	Z-Score distal aortic arch	Serum lactate level pre- stent (mg/dL; URL: 18 mg/dL)	Serum creatinine pre-stent (mg/dL; URL 0.9 mg/dL)	Arterial pH pre-stent	LV function pre-stent	LV-function post stent
1	Female, 11 days, 3.4 kg	Shone complex, CoA, left persisting superior vena cava, perinatal asphyxia, generalized convulsion, Goldenhar syndrome	Endotracheal intubation	Unclear neurologic state with repeated generalized convulsions. external center. no cardiac surgery	NA	NA	NA	NA	NA	NA	NA
2	Male, 6 days, 3.0 kg	CoA, hypoplastic arch, bicuspid aortic valve, borderline left ventricle, left persisting superior vena cava, endocardial fibroealstosis	No	Cerebral hemorrhage grade II, biventricular dysfunction	4	−4	27	0.56	7.34	Severely impaired	Markedly improved
3	Female, 10 days, 3.0 kg	CoA, hypoplastic arch	Nasal high-flow	Neonatal sepsis, impaired lower body perfusion	3.5	−3.2	12	0.4	7.36	Normal LV function	Normal LV function
4	Female, 8 days, 4.1 kg	CoA, hypoplastic arch	Endotracheal intubation	Cardiogenic shock	4	−4.4	65	0.55	7.33	Severely impaired	Moderately impaired
5	Female, 22 days, 2.5 kg	CoA, perimembraneous VSD	Nasal high flow	Severe biventricular dysfunction, preterm, low weight	4.5	−1.7	34	0.53	7.3	Severely impaired	Normal LV function
6	Male, 2 days, 2.8 kg	CoA, hypoplastic arch, bicuspid aortic valve, mitral stenosis, muscular VSDs	No	Cardiogenic shock	3	−6.1	28	0.83	7.35	Severely impaired	Normal LV function
7	Male, 22 days, 3.9 kg	CoA	Endotracheal intubation	Cardiogenic shock	NA	NA	17	0.37	7.53	Severely impaired	Moderately impaired
8	Male, 28 days, 2.7 kg	CoA, atrioventricular septal defect	Endotracheal intubation	Complex anatomy. preterm, low weight, stent as bridging to corrective surgery	NA	NA	20	0.27	7.43	NA	Normal LV function
9	Female, 33 days, 3.4 kg	Shone complex, CoA	No	Complex anatomy, biventricular dysfunction, pulmonary arterial hypertension	5	−1.6	9	0.26	7.32	Moderately impaired	Normal LV function
10	Female, 3 days, 2.9 kg	CoA, hypoplastic arch, bicuspid aortic valve, mitral regurgitation	No	Cardiogenic shock. lactate acidosis	3.5	−4.7	28	0.96	7.43	Severely impaired	Normal LV function
11	Female, 19 days, 4.6 kg	CoA, borderline left ventricle, pulmonary hypertension	Endotracheal intubation	Acutely decompensated heart failure	6	−1.5	NA	0.29	7.46	NA	Normal LV function
12	Male, 2 days, 4.0 kg	CoA, bicuspid aortic valve, ASD, muscular VSD	NA	Neonatal sepsis, heart failure, external center, no cardiac surgery	NA	NA	NA	NA	NA	NA	NA
13	Female, 25 days, 5.0 kg	Shone complex, CoA, left persisting superior vena cava, VSD, ASD	No	Complex anatomy. cerebral hemorrhage grade II	5	−1.3	NA	0.26	7.28	Normal LV function	Normal LV function
14	Male, 18 days, 3.7 kg	CoA. Congenitally corrected transposition of the great arteries. VSD. Ebstein anomaly	Endotracheal intubation	Cardiogenic shock	3.4	−5.7	122	0.42	6.93	Severely impaired	Mildly improved
15	Male, 8 days, 3.0 kg	CoA, hypoplastic arch, bicuspid aortic valve, muscular VSD	No	Severely impaired LV function	NA	NA	9	0.27	7.26	Severely impaired	Mildly improved
16	Male, 36 days, 2.6 kg	CoA, hypoplastic arch, bicuspid aortic valve, VSD, Turner syndrome	No	Complex anatomy	3.4	−4.9	14	0.36	7.4	Normal LV function	Normal LV function
17	Female, 10 days, 2.7 kg	CoA, bicuspid aortic valve	No	Cerebral hemorrhage grade II, cardiogenic shock	3.5	−4.5	47	0.21	7.27	Normal LV function	Normal LV function
18	Male, 10 days, weight NA	CoA, hypoplastic arch, bicuspid aortic valve	NA	External center, no cardiac surgery	NA	NA	NA	NA	NA	NA	NA
19	Male, 32 days, 2.2 kg	CoA, bicuspid aortic valve, borderline left ventricle	No	Neonatal sepsis	4	−2.4	32	0.39	7.24	Normal LV function	Normal LV function
20	Female, 31 days, 2.9 kg	CoA, hypoplastic arch, large muscular VSD, ASD	Nasal high flow	Unclear neurologic state with repeated generalized convulsions, cardiopulmonary resuscitation of unclear cause	3.9	−6.5	13	0.8	7.3	Mildly impaired	Normal LV function
21	Female, 25 days, 3.4 kg	CoA, hypoplastic arch, bicuspid aortic valve, muscular VSD	Nasal high flow	Respiratory syncytial virus infection, severe respiratory impairment	4.3	−3.9	17	0.23	7.33	Mildly impaired	Normal LV function
22	Female, 12 days, 3.3 kg	CoA, large inlet-to-outlet VSD	Nasal high flow	Cardiogenic shock	5	−1.7	58	0.49	7.25	Severely impaired	Mildly impaired
23	Male, 34 days, 3.9 kg	CoA, hypoplastic arch, bicuspid aortic valve congenital diaphragmatic hernia with left lung hypoplasia	vvECMO. endotracheal intubation	Ongoing vvECMO therapy due to respiratory impairment	NA	NA	NA	0.28	7.3	Normal LV function	Normal LV function
24	Female, 10 days, 3.4 kg	CoA, hypoplastic arch, bicuspid aortic valve, multiple VSDs	Endotracheal intubation	Cardiogenic shock	4	−3.8	183	0.68	7.07	Severely impaired	Moderately impaired
25	Male, 22 days, 3.0 kg	CoA, diaphragmatic hernia with hypoplastic left lung	No	Necrotizing enterocolitis	5	−1.4	NA	NA	7.22	NA	Normal LV function
26	Female, 3 days, weight NA	CoA	NA	Cardiogenic shock. external center, no cardiac surgery	NA	NA	NA	NA	NA	NA	NA

Abbreviations: ASD, atrial septal defect; CoA, coarctation of the aorta; LV, left ventricle; NA, not available; URL, upper reference limit; VSD, ventricular septal defect; vvECMO, venovenous extracorporeal membrane oxygenation.

**Figure 1.**
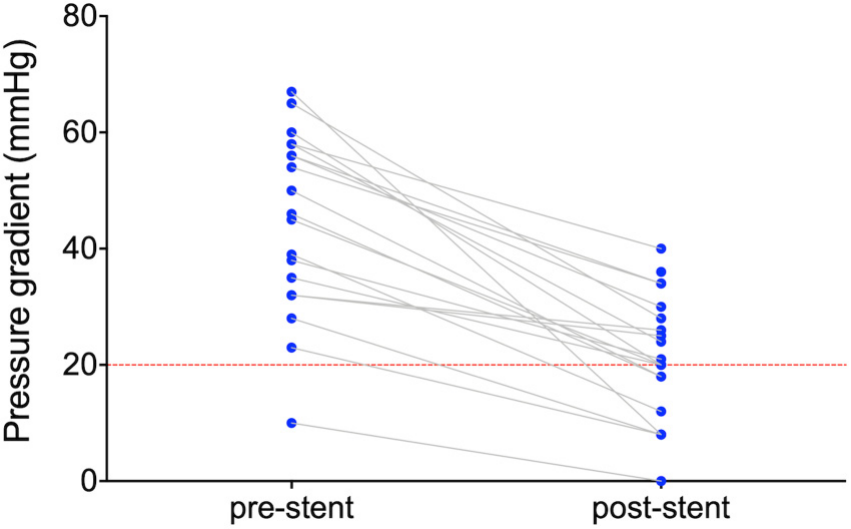
Pressure gradient over the coarctation region before and after stenting.

The median patient age at the time of stenting was 20 days (interquartile range (IQR) −33 days). Technical characteristics are shown in Table 2. Stenting resulted in a reduction of the pressure gradient in all patients. However, in eight patients (31%), a pressure gradient of more than 20 mm Hg remained after stenting (Figure 1). There were no procedure-associated complications and no mortality after the stenting procedures. In particular, there were no femoral artery occlusions or stenoses as documented via sonography at the time of surgery.

**Table 2.** Interventional Procedural Characteristics.

Parameter	Stented CoA patients (n = 26)
Age at initial stent implantation (d), median, IQR	20 (8-32)
Stent material, n (%)	
Bare metal coronary stent	25 (96)
Unknown	1 (4)
Stent diameter (mm), median, IQR	4.5 (4-6)
Stent length (mm), median, IQR	12 (11-16)
Femoral access route, n (%)	26 (100)

Abbreviations: d, days; IQR, interquartile range.

**Figure 2.**
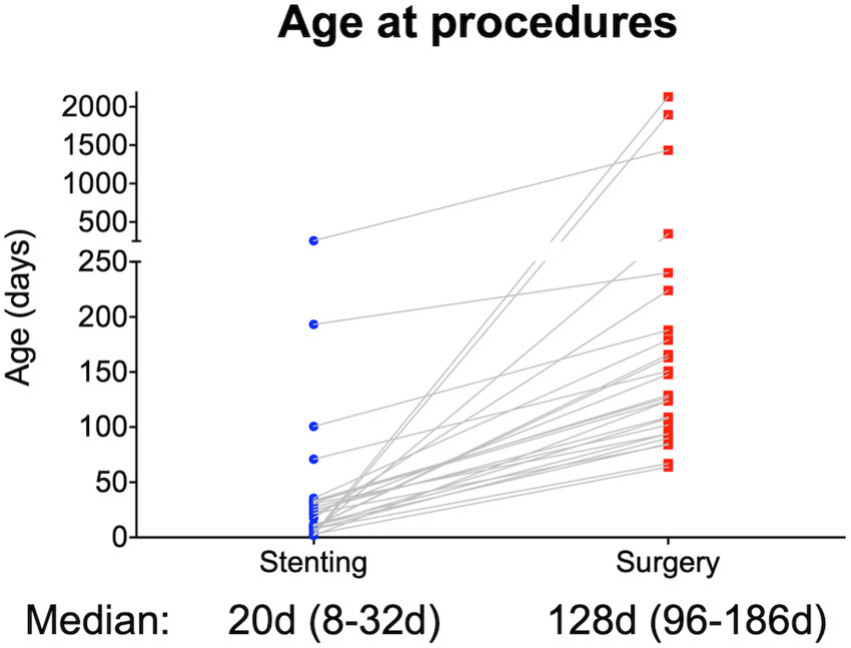
Age at stenting and surgical CoA repair. The median delay between stenting and surgical CoA repair was 92 days. CoA, coarctation of the aorta; d, days.

**Figure 3.**
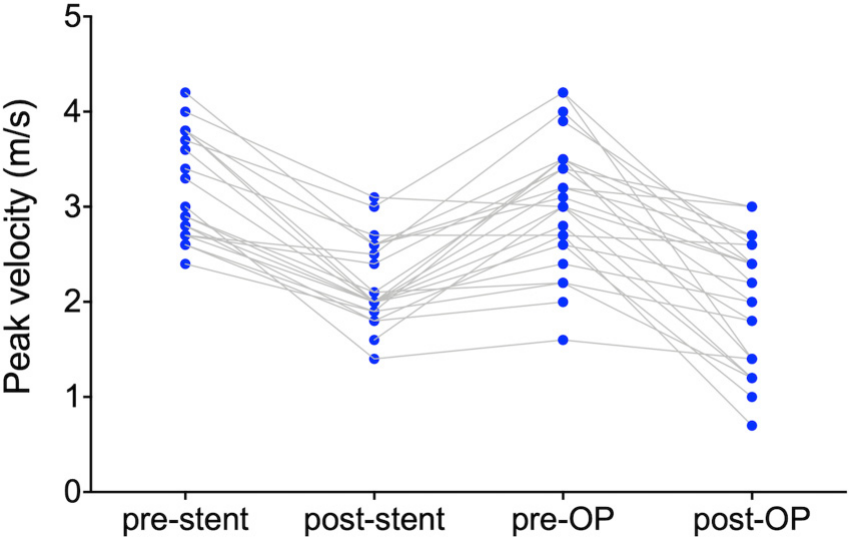
Peak velocity at the isthmus region before and after stenting and before and after surgical repair.

**Figure 4.**
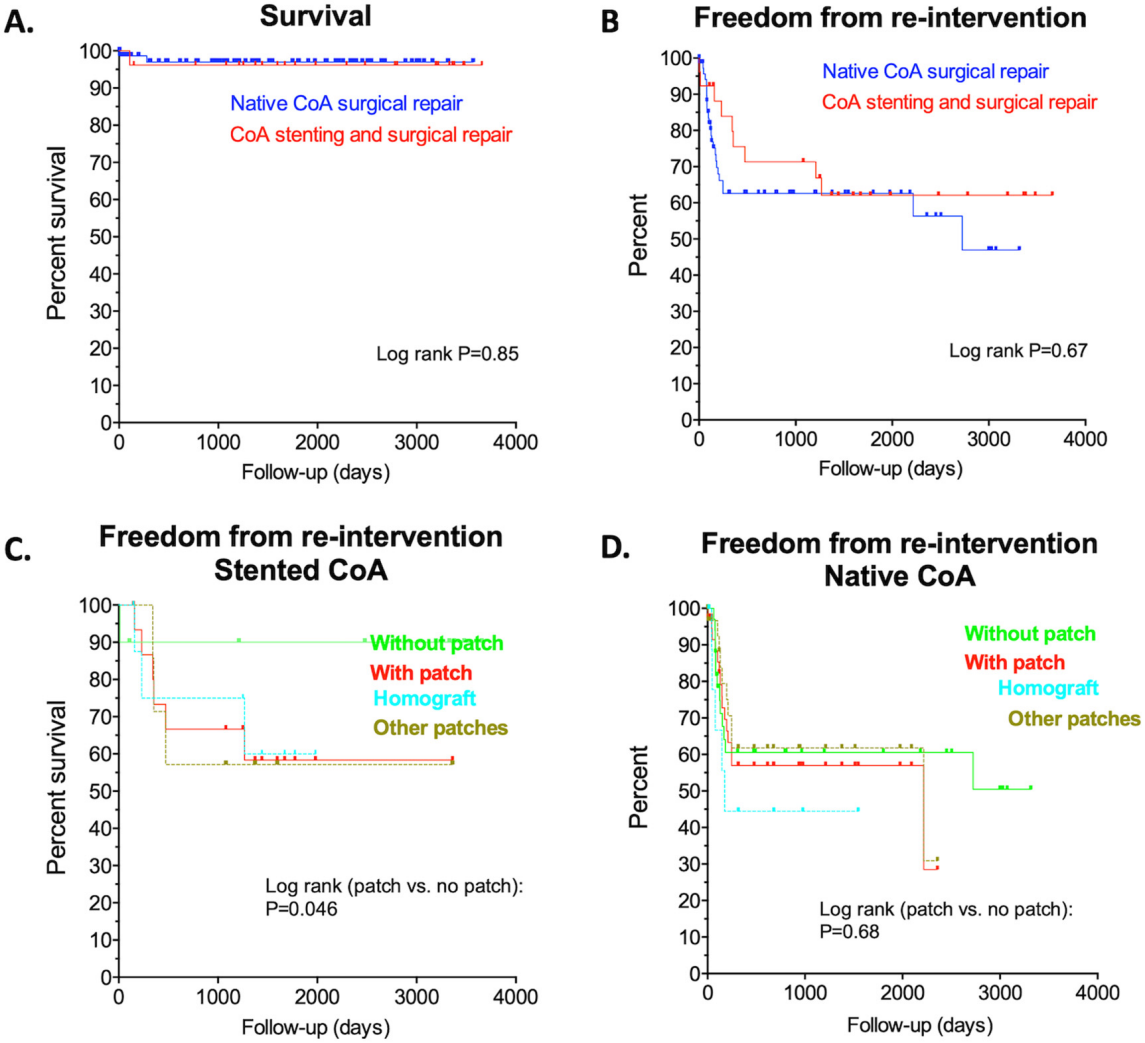
Long-term follow-up Kaplan Meier-estimations of (A) survival, (B) freedom from re-interventions after the staged approach or surgical repair of native CoA, (C) freedom from re-intervention according to surgical technique in stented CoA, and (D) freedom from re-intervention according to surgical technique in native CoA. CoA, coarctation of the aorta.

The median age at the time of surgical repair was 128 days (IQR 96-186 days) after a median waiting time of 92 days (IQR 75-151 days; Figure 2). Surgery was conducted over a median sternotomy in 25 patients (96%) using cardiopulmonary bypass and cardioplegic arrest (Table 2). In all four patients who were initially stented due to complex cardiac malformations (two patients with Shone complex, one patient with complete atrioventricular septal defect, one patient with aortic valve stenosis and ventricular septal defect), anatomical correction was achieved during the surgical procedure. In 16 patients (62%), a complex aortic reconstruction with the use of patch material (xenopericardial patch (n = 4); autologous pericardium (n = 2) or homograft material (n = 9)) was necessary. The median Z-score of the distal aortic arch diameter of these patients was −3.85 (IQR −4.6 to −1.7). In four patients, complete extraction of the stent material was not possible. The aortic peak velocity was reduced from preoperatively 3.0 m/s (IQR 2.6-3.5 m/s) to 2.1 m/s (IQR 1.4-2.6 m/s) postoperatively. Interestingly, the peak velocity was also reduced by the previous neonatal stenting but increased again until surgery (Figure 3). The 76 contemporary patients who underwent surgery of native CoA were younger and weighed less at the time of surgery (Table 3). They were treated more frequently using a lateral thoracotomy approach (32% vs 4%; *P* = .003) with corresponding differences in the use of CPB. The decision toward a median sternotomy approach was predominantly driven by the need for repair of additional cardiac lesions in the native patients. Contrarily, in the stented patients, a median sternotomy approach was predominantly chosen due to arch hypoplasia or the anticipated need for complex arch repair (Table 3). The use of patch material was not different for native patients compared with the stented patients (54% vs 62%; *P* = .65). The rates of postoperative complications (bleeding, recurrent laryngeal nerve or phrenic nerve paralysis) were comparable in both groups (Table 3). One previously stented patient died 33 days postoperatively (3.8%). This patient underwent complex reconstruction with end-to-side-anastomosis of the aorta and concomitant repair of a complete atrioventricular septal defect. The patient died from septic multi-organ failure due to Staphylococcus aureus sepsis. In the native CoA group, there were two in-hospital mortalities (2.6%), both due to left ventricular failure postoperatively. The further follow-up of the stented patients (median follow up 5.2 years after the stenting procedure) and the native CoA patients (median follow up 3.1 year) showed no further mortality (*P* = .78; Figure 4A).

**Table 3.** Surgical Strategy, Adverse Outcomes, and Re-interventions.

Parameter	Stented CoA patients (n = 26)	Native CoA patients (n = 76)	*P* value
Age at surgery (d), median, IQR	128 (96-186)	21 (10-29)	.002
Body weight (kg), median, IQR	5.6 (4.5-6.5)	3.6 (3.1-3.8)	.045
Arch hypoplasia, n (%)	11/20 (55)	41/71 (57)	.58
Surgical strategy			
Surgical access, n (%)			
Median sternotomy	25 (96)	52 (68)	
Left lateral thoracotomy	1 (4)	24 (32)	.003
Reason for median sternotomy approach, n (%)			
Arch hypoplasia or expected extended repair	21/25 (84)	24/52 (46)	.0026
Repair of additional defects	1/25 (4.0)	2/52 (3.8)	1.00
Both	3/25 (12)	26/52 (50)	.0012
Use of extracorporeal circulation, n (%)	25 (96)	52 (68)	.003
Technique of aortic repair, n (%)			
End-to-end-anastomosis	1 (3.8)	2 (2.6)	1.00
Extended end-to-end-anastomosis	4 (15)	25 (33)	.13
Patch aortoplasty	17 (65)	44 (58)	.64
Ascendo-descendostomy	4 (15)	5 (6.6)	.23
Use of patch material, n (%)	16 (62)	41 (54)	.65
Homograft patch, n (%)	9/16 (56)	10/41 (24)	.03
Other patch material, n (%)	6/16 (38)	31/41 (76)	.03
Adverse outcomes			
Re-exploration for bleeding, n (%)	0	3 (3.9)	.57
Recurrent laryngeal nerve paralysis, n (%)	1 (3.8)	5 (6.6)	1.00
Phrenic nerve paralysis, n (%)	2 (7.6)	8 (11)	1.00
In-hospital mortality, n (%)	1 (3.8)	2 (2.6)	1.00
Re-interventions due to re-coarctation			
Duration of follow-up (years), median	5.2	3.1	
Time to re-intervention (days), median, IQR	288 (179-341)	127 (80-181)	.0025
Patients with re-interventions, n (%)	10 (40)	28 (38)	.67^a^
Re-Intervention before surgical repair	4/10 (40)		
Re-Intervention after surgical repair	4/10 (40)	28/28 (100)	
Re-interventions before and after surgical repair	2/10 (20)		

Abbreviations CoA, coarctation of the aorta.

^a^ *P* value resulting from Kaplan-Meier analysis.

However, restenoses requiring re-interventions occurred in both groups: in the staged approach, there occurred re-interventions in a total of ten patients (40%). Between the initial stenting procedure and the surgical correction, balloon angioplasties of the stent were necessary for three patients and additional stent placement was performed in another three patients. After surgical repair, a total of six patients (two of them already had a re-intervention between the stenting and the surgical correction) needed surgical (n = 4) or catheter (n = 2) re-interventions by means of balloon dilatation. Two of the four patients who needed surgical re-intervention were primarily re-intervened by balloon dilatation of the re-coarctation, one of them with stent implantation. In the native CoA group, a total of 28 patients (38%) needed re-interventions (surgical n = 8, interventional n = 20; Table 3), resulting in a comparable re-intervention rate as in the stented patients (Figure 4B; *P* = .67). Of these patients, 20/28 (71%) had the CoA repair with use of median sternotomy and cardiopulmonary bypass (whole native CoA group: 52/76; *P* = .82). In the 20 patients who underwent median sternotomy, arch hypoplasia was present in eight patients (40%) and arch hypoplasia with additional cardiac defects warranting repair were present in 12 patients (60%). These rates are also comparable to the rates in the whole group (arch hypoplasia: 46%, arch hypoplasia and additional defects: 50%, Table 3). The re-intervention rates in the staged approach differed statistically significantly depending on the reconstruction technique applied (reconstruction without patch material vs reconstruction with patch material; *P* = .046; Figure 4C). Interestingly, in the staged approach group re-interventions after the surgical repair only occurred in patients who received aortic reconstruction with patch material with no obvious difference between patients who received homograft patches versus other patches. Of note, in the native CoA patients, the re-intervention rates did not differ significantly between patients who were treated with or without the use of patch material, respectively (*P* = .68; Figure 4D). Re-interventions occurred after a median of 288 days (IQR 179-341 days) postoperatively in the stented patients and significantly earlier in native CoA patients (127 days; IQR 80-181; *P* = .0024, Table 3).

## Comment

The main result of this study is that neonates presenting in critical condition with CoA can be treated with low mortality using a staged approach consisting of initial palliative stent implantation followed by surgical CoA repair. The mortality rate (3.8%) in the investigated patient population was lower than the mortality rates described by McGuinnes et al (7%) or Bacha et al (5.6%) for primary surgical repair in high-risk patients with CoA.^8,17^ However, it should be noted that the criteria for “high-risk patients” were different in the cited studies limiting the comparability of the results with the results of this study. Concerning the staged approach with stenting and subsequent surgical repair, other groups have described outcomes in smaller patient populations with similarly low perioperative mortality (0%-13%).^11,12,18^

Until now there are no data on the optimal timing of surgical stent removal after the initial stenting of CoA. Our data suggest acute CoA relief after stenting as the peak velocity and pressure gradient are reduced. However, in most patients, the velocity had increased again by the time of surgery, probably owing to stent ingrowth and growth of the patients. Although earlier surgical repair might be favored in order to avoid these effects by the time of surgical removal, a longer recovery period might be favorable because the patients would undergo surgery under more stable conditions. In our experience, for a full recovery from cardiogenic shock and associated renal function or hepatic function impairments or from severe neonatal infections like necrotizing enterocolitis, a delay of at least two months seems desirable. The surgical data from this population suggest that complete stent removal is challenging after this time. However, the risk imposed by surgically more difficult aortic reconstruction has to be weighed against the risk caused by a still incompletely recovered organism after neonatal critical illness. As Fouilloux et al pointed out, some centers prefer early removal of the stent within 14 days post-interventionally.^19^ However, in our experience, recovery of those children treated with stenting was not sufficiently complete at that time point so that surgery at this early stage seems still risky. In summary, we consider a surgical CoA repair with stent removal approximately two to three months after stenting to be a good compromise in most of the cases.

Another possible strategy for initial palliation could be balloon dilatation without a stent which is also preferred by some groups. This strategy would eliminate the need for later stent removal, possibly reducing the complexity of surgical repair. However, there are three aspects that, in our view, limit this strategy: First, due to elastic recoil of ductal tissue in the CoA region, the result of balloon angioplasty is not as predictable as that achieved by stent implantation. Second, the result is likely to be not as permanent which might lead to suboptimal palliation and impaired recovery of the patient. Third, vascular complications associated with balloon angioplasty including aortic dissection and late aneurysm formation are feared complications of CoA balloon angioplasty.^20^ Of note, for this particular clinical context where a durability of the interventional therapy of no more than weeks to months is needed, there exist no comparative data on balloon versus stent angioplasty. These data would be desirable to obtain in order to define the optimal strategy.

From a logistic point of view, some of the patients in this study population were initially treated with stent placement in external hospitals without congenital cardiac surgery structures in place. This fact might distort the indications for stenting as the local nonavailability of cardiac surgical care might lead to a more liberal indication for stenting in order to avoid the risk of an interhospital transfer of a critically ill neonate.

From a technical point of view, we consider some aspects during the stenting to be critical for an as uncomplicated as possible further therapeutic course, particularly during the subsequent surgical therapy. First, the stent should be chosen as short as possible in order to minimize the amount of aortic tissue consumed by ingrowth of the stent that needs to be removed during surgery. For the same purpose, the stent should not be overdilated. Overexpansion of the stent would optimize proximal and distal apposition of the stent but would also lead to a firmer stent ingrowth over its full length.

The overall utility of this approach compared with a primary neonatal surgical repair is not identifiable in this retrospective setting. In order to bring the outcomes into context, the outcomes of neonates who were not classified as high-risk patients and who underwent primary surgical repair during the same time period at our institution were also analyzed. Here, the stented and native CoA patients showed similar perioperative complication and mortality rates. This suggests that the bridging with palliative stenting toward surgical repair at an older age and safer circumstances might be helpful for critically ill neonates with CoA.

The rate of re-intervention in our study population was 40% for the patients who underwent the staged approach (including re-interventions before surgical repair) and 38% in the native CoA population over the follow-up interval. Although the re-intervention rates in the staged approach need to be seen in the context of two procedures, the re-intervention rate in the native CoA group is unexpected, also given the lower rate of patch material use in the native CoA group. However, the data on the role of patch aortoplasty as a risk factor for recurrent CoA are conflicting, as discussed in a recent systematic review by Dias et al.^21^ Indeed, in the native group, there was no difference in re-intervention rates between patients who were treated with or without patch material, respectively. Contrarily, re-interventions after the surgical repair in the stented patients only occurred in patients who needed more complex CoA repair with the use of patch material. These re-intervention rates need to be considered rather high when compared with previous studies reporting about surgical repair of native CoA.^22,23^ However, the recent review by Dias et al again points out that young age (to a small extent) and low weight (<2.5 kg) at surgical repair are risk factors for re-coarctation.^21^ In our native CoA collective, the median age at operation was 21 days which might have increased the risk of re-coarctation. However, the median weight of these patients was 3.6 kg and might as such not predispose to re-coarctation. Additionally, general complexity of the primary surgery as reflected by the presence of arch hypoplasia and/or additional cardiac defects did not seem to predispose to re-interventions in the native CoA patients. In the stented CoA group, the children underwent surgery at an older age and re-coarctations seemed to be rather triggered by more complex reconstruction techniques.

There are some limitations of this study that need to be mentioned. It is a retrospective single-center analysis with all its methodologically inherent limitations. The sample size is small, disabling any confirmatory conclusions. As stated above, the indications for stenting, particularly if conducted in external centers without cardiac surgical structures might have been inconsistent and were only traceable according to the institutional records.

Nevertheless, the data from this study suggest that stenting followed by surgical CoA repair, although probably increasing the technical complexity of subsequent surgical repair with considerable re-intervention rates, might be effective to bridge high-risk neonates to safer conditions for surgical repair.
